# Are Nutrition Knowledge, Attitudes, and Beliefs Associated with Obesity among Low-Income Hispanic and African American Women Caretakers?

**DOI:** 10.1155/2013/123901

**Published:** 2013-05-29

**Authors:** Irene Acheampong, Lauren Haldeman

**Affiliations:** Department of Nutrition, University of North Carolina, Greensboro, 319 College Avenue, 318 Stone Building, Greensboro, NC 27412, USA

## Abstract

The purposes of this descriptive study were to (1) describe nutrition knowledge, attitudes, beliefs (KAB), and self-efficacy among low-income African American and Hispanic women; (2) identify the associations these variables have on diet quality and weight status; (3) identify barriers to healthy eating. Data from three separate studies were combined and analyzed. The total sample included African Americans (*N* = 92) and Hispanics (*N* = 272). Descriptive statistics and bivariate analyses were used to identify associations between KAB and body mass index (BMI) and diet quality. The majority of African Americans had good knowledge in nutrition while Hispanics had fair knowledge. Attitudes toward eating a healthy diet were significantly associated with high fiber intake among African Americans and low fat consumption among Hispanics. A computed KAB score showed no significant relation to individuals' weight status or diet quality. However, attitudes and beliefs about healthy foods strongly correlated with participants' weight or diet consumption among Hispanics. The most common barrier to consuming a healthy diet reported by both groups was the cost of healthy foods. It is therefore recommended to address these variables when addressing obesity and poor dietary intake among low-income minority groups.

## 1. Introduction

Over a three-year span, the Centers for Disease Control and Prevention (CDC) recorded that African Americans and Hispanics, respectively, had a 51% and a 21% greater prevalence of obesity than Caucasians [1]. Statistics show that the risk of developing diabetes among African Americans and Latinos is twice that of Caucasians [2]. Similarly, the prevalence of cardiovascular disease and other weight-related health issues is higher among minority groups [2]. According to the US Census Bureau [3], the two largest minority groups in the US are Hispanics and African Americans; it is estimated that 52 million (16.7%) of the total US population are Hispanics and 40.8 million (13.1%) are African Americans. Compared to African Americans, Hispanics have higher health risks mainly because of limited English proficiency and less access to basic health care needs [4]. The CDC reported that Hispanics residing in the US are twice as likely to die from diabetes [5] than non-Hispanics.

It is well documented that minorities are particularly prone to health disparities [6, 7]. Though the causes of health disparity are still being debated [6, 8], it is believed that low socioeconomic status (SES) plays a significant role mainly among minorities and immigrants [9]. This is of particular importance as in the US, African Americans, Native Americans, Asian Americans, and Latinos tend to be overrepresented among people with low SES [10]. Low SES has been correlated to poor health [11] which consequently leads to a higher rate of diabetes, cardiovascular diseases and other obesity-related illnesses among African Americans and Hispanics [6]. This can be attributed, in large part, to certain dietary behaviors and factors such as knowledge, beliefs, and perceptions about nutrition and health.

A number of studies have established that an improvement in nutrition knowledge is an important tool to stimulate dietary behavior that will promote healthy weight in all generations [12, 13]. It has also been shown that increasing one's knowledge in nutrition improves attitudes, beliefs, and self-efficacy towards the consumption of a healthy diet and a possible increase in physical activity [14, 15]. Therefore, it is important to examine the relationship between nutrition knowledge and the consumption of a healthy diet [16–18] in order to improve the health status specifically among minority groups.

Though some solutions, such as food assistance programs, have been proposed and implemented to ensure a healthy diet among low-income earners [19, 20], many of these solutions need to be localized in order to target specific populations. It is evident that solutions that may work for a particular group of people may not be helpful for others due to factors such as the level of education, cultural beliefs, financial status, and different ethnic food consumption. Thus, to better tailor nutrition interventions to a specific group, it is important to understand the differences that exist between the two largest minority groups.

A few studies have focused on the differences in nutritional knowledge, attitudes, beliefs (KAB), and self-efficacy between African American and Hispanic groups [21, 22] and the possible influence they may have on weight status. The purpose of this study was to describe nutrition knowledge, attitudes, beliefs (KAB), and self-efficacy specifically among low-income African American and Hispanic women residing in North Carolina, who are caretakers of children under the age of twelve. Caregivers are of particular importance due to their unique needs and great impact on the quality of dietary intake in children and members of the household in general [23]. The study addressed differences in food security status, BMI, diet intake, and KAB and examined associations of BMI and KAB between the two ethnic groups.

## 2. Materials and Methods

Data used were from three previous studies conducted in Guilford and Chatham counties in North Carolina over a period of five years. Three data sets obtained from the studies were combined, cleaned, and merged on 298 common variables. They consisted of the nutrition knowledge, attitudes, and behaviors of rural (*N* = 119) and urban (*N* = 166) Hispanics [24] and urban African American women (*N* = 100) [25]. The studies examined the relationship between caretakers and their food choices.

### 2.1. Study Participants

Convenience samples of participants in all three studies were recruited from community agencies. Selection criteria included primary caretakers of children <12 years of age, main household food shoppers and meal preparers, Hispanic or African American, and low income. In-home interviews were conducted by trained interviewers. All participants were asked questions regarding food security status, diet quality, nutrition knowledge, attitudes, behaviors, beliefs, and self-efficacy. Heights and weights were also measured. Specifics regarding data collection for these studies are available in previous publications [24, 25]. Due to missing data the total sample size for the current study was 364, with 92 African American women and 272 Hispanics.

### 2.2. Measures

The CDC's BMI classification was used; underweight and normal weight categories were combined into one group due to the low number of underweight individuals.

#### 2.2.1. Variable Definitions and Scores

Variables were defined and additive scores were developed for all variables used in the data analyses process. The following section defines all variables and statistical analyses used to address this study's research questions.

*Nutrition Knowledge*. Participant's nutrition knowledge was evaluated using a face and content validated 20-item questionnaire. A score of one was awarded to questions answered correctly, and a zero was allocated to a wrong answer or a refusal/do not know response. Questions regarding good sources of saturated fat, calcium, iron, and fiber were included. A total of 20 points were possible. Individuals with ≤5 correct answers were considered to have a poor knowledge in nutrition, while those with 16 or more correct answers were considered to have an excellent knowledge in nutrition. Scores ≥6 but ≤10 were considered to be fair, and participants with ≥11 but ≤15 had good nutrition knowledge. These cutoffs were based on the distribution of the data.

*Attitudes Score*. To assess participants' attitudes towards certain foods, questions were asked regarding the importance of eating specific healthy foods. Participants who affirmed that it was important to eat fruits and vegetables, fiber, low fat and low sugar diets, and those who choose and eat a healthy diet, were assigned a score of 1, any other response (no, no response, and i do not know) was assigned a zero. The total possible score was 5.

*Belief Score*. Belief was measured based on a single variable: “healthy foods will help keep one healthy.” Participants with a positive nutrition belief that healthy foods will help keep them healthy were awarded a score of 1, all other responses were assigned a zero.

*Knowledge, Attitude, and Belief (KAB) Score*. Scores obtained from the nutrition knowledge, attitude, and belief questions were added to compute a total KAB score. The highest possible total KAB score was 26 and 0 was the lowest. The scores were divided into groups of six and categorized into poor (scores ≤ 5), fair (scores 6 to 12), good (scores 13 to 19), and excellent (scores ≥ 20). These cutoffs were based on the distribution of the data.

*Barriers and Self-Efficacy*. Barriers to preparing or consuming a healthy diet were determined primarily by asking the participant if it was difficult to eat a healthy diet. Participants that responded yes were further asked to state why it was difficult to eat a healthy diet. Self-efficacy was assessed by asking participants how confident they were in choosing healthy foods at the grocery stores, preparing healthy foods for their family, and selecting healthy snacks for their children.

*Food Security*. Assessment of food security status was conducted using the 18-item US Food Security Survey Module. Food security categories as defined by the USDA are food secure, marginal food secure, low food secure, and very low food secure [26]. Based on the distribution of the responses, food secure was combined with marginal food secure to form one category, and the responses were classified into 3 categories of food security status according to the US Food Security Scale. 

*Diet Intake*. A food frequency questionnaire was administered, and dietary intake was measured by the consumption of certain specific foods, such as tortilla, bread, rice, fruits, and vegetables. These foods were then categorized into the five main food groups.  Table 1 illustrates the food groups with the individual food items and the serving sizes, as recommended by MyPlate/Food Guide Pyramid, were used in determining if participants met or did not meet the food recommendations. Individuals that met the least serving size per day of the group were considered to have met the daily requirement for that specific food group. For instance, if a respondent reported consuming 2 servings of fruits per day then they were determined to have met the daily recommendation for fruit. Conversely, if a respondent reported consuming only 5 servings of grains they had not met the recommendation for the grain group. 

*Diet Quality*. Mean dietary quality was calculated by summing up, at least, four food groups and dividing the value by 5. This analysis was conducted to investigate if the population sampled were meeting the recommendations from the five food groups. The mean number of servings was calculated by summing up the total number of servings for a particular food group consumed by the ethnic group and dividing it by the total number of participants in that group. For example, the mean number of servings for dairy among African Americans was computed by adding the number of dairy servings consumed by each participant and dividing the sum by 92. This gave a mean dairy intake of 1.58 servings per day.

### 2.3. Data Analyses

SPSS version 18.0 was used to conduct all analyses. Descriptive statistics were used to describe the Hispanic and African American samples in terms of sociodemographics and variables of interest. Student's *t*-tests and ANOVA were conducted to compare key sociodemographic variables on nutritional knowledge, attitudes, beliefs, self-efficacy, barriers, and food security between the two ethnic groups. Statistical significance was denoted by a *P* value <0.05.

## 3. Results

### 3.1. Sample Characteristics of African American and Hispanic Women

#### 3.1.1. Demographic Variables

From the demographic variables analyzed, more African Americans (87%) considered themselves as the head of household, when compared to the Hispanic population (18.4%). High participation in the WIC program was observed among the Hispanic families (98.9%); however, very few (20.3%) benefited from the Supplemental Nutrition Assistance Program (SNAP) (food stamps). More African Americans participated in SNAP (84.8%) than the WIC program (71.6%). Additionally, African Americans were more likely to earn a higher income and have a smaller family size than the Hispanic group. In terms of transportation, most participants had easy access to a car when the need arose.  Table 2 illustrates the above characteristics.

#### 3.1.2. Food Security

As shown in Table 3, the majority of African Americans were in the food secure/marginal food secure group, while most Hispanics experienced low food security. African Americans, however, had more than double the percentage of households in the very low security category than Hispanics.

#### 3.1.3. Weight Status

In terms of weight status, the mean BMI for African American women was 33.1 ± 8.8 kg/m^2^ and 28.9 ± 5.6 kg/m^2^ for the Hispanic women. Between the two ethnic groups, a higher percentage of African Americans, than Hispanics, were obese (61.5% versus 40.3%, resp.) (Table 4).

#### 3.1.4. KAB Valuables, Barriers, and Self Efficacy Variables

With regard to nutrition knowledge, most African American women had good nutrition knowledge, while the highest percentage of Hispanic women had a fair knowledge.

In terms of self-efficacy, African Americans versus Hispanics were significantly more likely to be very or somewhat confident in choosing (97.8% versus 69.5%), preparing (96.7% versus 76.5%), and selecting (98.9% versus 77.2%) healthy foods for their families.

Among African American participants who stated that eating a healthy diet was difficult, their most common reported barriers included the cost of the foods, the time it takes to prepare a healthy meal, and difficulty in finding healthy foods. The least common barrier was the ability to read food labels. Barriers specific to the Hispanic group included lack of knowledge for purchasing healthy foods and difficulty in reading food labels.

More Hispanics (29.4%), than African Americans (18.5%), described their diet as healthy. Cross tabulation showed that the perception of fiber as an important component of the diet was significantly related to ethnicity (*P* = 0.016). The majority of African Americans agreed that fiber was an important part of the diet. Responses to the importance of fiber and low fat intake were the only variables, pertaining to attitudes, that were significantly different between ethnicities (*P* = 0.016 and 0.007, resp.). When compared to African Americans, more Hispanic women stated that it was more important to eat a diet low in fat.

Overall, 95.1% stated that healthy food choices will help keep one healthy. Of this percentage, 90.2% were African Americans and 96.7% were Hispanic women.

#### 3.1.5. KAB Score

In terms of the computed nutrition KAB score, 0%, 18.5%, 63%, and 18.5% of African Americans had a poor, fair, good, and excellent KAB level, respectively. Among Hispanics, 1.8%, 26.5%, 54.8%, and 16.9% had a poor, fair, good, and excellent KAB level, respectively. It was noted that more than half of the obese respondents had “good” KAB score. That is, these participants have good knowledge and positive attitudes and beliefs regarding nutrition.

#### 3.1.6. Dietary Intake

Regarding dietary intake, the average reported consumptions of dairy, fruits, meat, and grains were higher among Hispanics. African Americans were twice as likely, than Hispanics, not to meet any of the dietary recommendations. The majority of Hispanics met at least one dietary recommendation. Hispanics consumed a higher amount of grains, but African Americans consumed more vegetables. Participants who experienced low food security were more likely to meet more of the recommended dietary intake (*P* = 0.003). Among African Americans, a significant difference was observed in fruit consumption (*P* = 0.023) and food security. High fruit consumption was observed among food secure/marginal food secure individuals. One-way ANOVA indicated that the mean dietary intake significantly differed by ethnicity (*P* < 0.001). Hispanics had a higher mean intake than African Americans.  Table 5 illustrates the mean number of servings per food group with their standard deviations (SDs) and range.

#### 3.1.7. Relationships between Variables

Among African Americans, weight status significantly correlated with food security and attitude towards eating a healthy diet. Those that were food secure versus food insecure were more likely to be overweight/obese (45.0 versus 33.9; *P* = 0.014, resp.). Those in the under/normal weight versus those in the overweight/obese category were more likely to have more positive attitudes (earned a 4 on the attitude scale) about eating healthy (42.1 versus 27.8; *P* = 0.013, resp.). These associations were not significant among the Hispanic group. For both ethnicities, food security significantly correlated with dietary intake. Among African Americans those that were food secure were more likely to have better diet quality compared to those that were food insecure (*P* = 0.036). Conversely, food insecure Hispanics were more likely than food secure Hispanics to have a better diet quality (*P* = 0.001). Among the Hispanic group, a significant relationship was observed between nutritional knowledge and attitude (*P* < 0.001). Participants with good/excellent nutrition knowledge versus poor/fair nutrition knowledge were significantly more likely to earn the highest attitude score and thus have the most positive attitudes about eating healthy (59.4 vs. 40.6; *P* < 0.001, resp.). Additionally, among the Hispanic population, the belief that healthy foods will keep one healthy was significantly associated with a better diet quality (*P* = 0.008).

## 4. Discussion

The purpose of this study was to describe nutrition knowledge, attitudes, beliefs (KAB), self-efficacy, and barriers among low-income African American and Hispanic women caretakers of young children and how these variables influence their diet quality and weight status. Demographic statistics show that in North Carolina, almost half of the African American women (48.9%) and 17.4% of Hispanic females serve as the head of household [27]. In the present study, monthly income differed between ethnicities. On average, the African Americans sampled earned almost twice as much as Hispanic families. This finding is similar to the reported state income by race [28]. A higher percentage of Hispanics, than African American women, participated in the WIC program. SNAP was, however, less utilized by the Hispanic population. This could be due to the eligibility requirements set for SNAP [29].

One factor that affects food intake is the level of food security. In 2008, the national rates for low and very low food security among Hispanic households were 26.9% and 8.8%, respectively [30]. The percentage of households that experienced very low food security, among the population studied, was slightly lower than the national rate. However, a relatively higher percentage (77.2%) experienced low food security as compared to the national rate. Among African Americans, the rate of low food security in the current study was slightly lower than the national rate of 25.7%. Food security was significantly associated with ethnicity, similar to several research studies [31–33]. Individuals in the obese category were more likely to experience very low food security as compared to individuals who are normal or over weight. This association of low food security with obesity has also been observed in previous research [10, 17, 18, 34, 35], and one proposed explanation of this paradox is that low food secure families tend to purchase high caloric meals in order to stretch the value of their low income [35].

Among the two ethnicities studied, there were some slight differences in beliefs, attitudes, self-efficacy, and barriers to healthy eating. More Hispanics, than African Americans, believed that making healthy food choices will help keep them healthy. Despite this belief, only a few women described their diet as healthy. It was observed that African Americans had higher healthy eating self-efficacy than Hispanics. A greater number of African Americans expressed a high confidence level regarding healthy eating and making healthy food choices for their families. This could be due to the years of exposure to healthy American foods [36]. The language barrier, among Hispanics, could be a factor affecting their self-efficacy of healthy eating; as most of the food labels, commercials, and educational materials on healthy foods are in English. One unique barrier to the Hispanic women was difficulty in reading food labels. This could explain why Hispanics have been reported to use food labels less than non-Hispanic groups [37]. The use of food labels has been positively associated with nutrition knowledge [38, 39], and research studies show that even though most Hispanics are familiar with the food labels, only a few are confident in using them [40]. The two most common barriers to healthy eating among African Americans were the length of preparation and cost of healthy foods. Among Hispanic families, the cost and difficulty in getting healthy foods that their families would like were identified as primary barriers. These reported barriers are in line with previous studies [22, 41]. The least identified barrier for both ethnicities was the dislike of healthy foods.

The majority of the study sample agreed that fruits and vegetables are vital components of a healthy diet; however, only 62.1% believed that fiber was important. This may illustrate the low nutrition knowledge of the participants concerning the sources of fiber and its benefits. The perception that fiber is an important component of the diet was significantly related to ethnicity. More African Americans, than Hispanic women, stated that fiber is important. It has been noted that Hispanic women in the US, on average, consume about 14 g of fiber/day [42] which is much lower than the recommendation of 25-25 g/day. Previous research shows that though 78.1% of Hispanics have heard about fiber, only 13.4% are able to recognize the definition of fiber [40]. Thus, there is a need for nutrition education in the area of the sources and benefits of fiber among the Hispanic population.

In terms of daily food intake, grains were consumed more than any other food group, while vegetables were consumed the least. Among the two ethnicities, the average consumption of dairy, fruits, meat, and grains was higher among Hispanics than African Americans. Similar findings of ethnic differences in dietary intake have been documented in previous research [43]. The fact that vegetable intake was lower among Hispanic women than African Americans is contradictory to previous studies [42, 44]. However, this may be explained by the high consumption of potatoes, which was classified as a vegetable within the survey. The number of dietary recommendations met was significantly associated with ethnicity. In agreement with previous studies [45], our results showed that the Hispanic population met more dietary recommendations than African Americans. A majority of African Americans had good nutrition knowledge, while most of the Hispanics had only a fair knowledge about nutrition. As observed in previous research [46], the current study did not observe any significant relationship between participant's knowledge in nutrition and their BMI or their diet quality. This finding was contrary to other research [16–18] which reported higher BMI levels being associated with poor nutrition knowledge. In the present study, 89.7% of overweight/obese individuals had fair, good, or excellent nutrition knowledge. This implies that, although overweight/obese individuals have good nutrition knowledge, they may not be utilizing it to make healthy food choices. Another possibility is that the knowledge being shared with this population is not practical nor is it tailored specifically to obese mothers. Kreuter et al. [21] noted that the method of delivering nutrition education is as equally important as the message being conveyed. The total combination of participants' knowledge, attitude, and belief scores (KAB) did not show any significant association with BMI or diet quality; however, in both ethnicities, overweight/obese individuals had higher KAB scores.

## 5. Conclusions

Among Hispanics, nutrition knowledge was associated with attitude. Belief was related to diet quality, and food security was related to BMI and mean intake of food. Among African Americans, attitude was significantly related to BMI and food security level to diet intake. Among African Americans, the majority that had good KAB scores did not meet any of the dietary recommendations. However, within the Hispanic population, those that had good KAB scores met at most 2 of their dietary recommendations. The majority of both ethnic groups that had good KAB scores were obese.

## 6. Research Limitations

This cross-sectional study had several limitations. The study sample was a convenient nonrandom sample, and the results therefore cannot be generalized to other groups. Score ranges for nutrition knowledge and KAB categories were not standardized nor tested. Also, diet quality did not include fats, oils, and sweets nor did it consider the total calories taken in or expended. Additionally, because the diet analysis and that of nutrition knowledge are based on the former Food Guide Pyramid (FGP), food groupings were determined based on those established based on the FGP. Thus, grain-based snacks like potato chips, nachos, and cookies were placed in the grain group and calculated as such in the KAB score. Lastly, these were secondary data; thus, we had no control how data were collected or measured.

## 7. Future Implications

The variation in diet, KAB, and self-efficacy between the two ethnic groups supports the need for nutrition educators to acknowledge these differences and focus on group specific needs as they relate to dietary intake or BMI. For instance, among Hispanics, beliefs influence food intake. This was not evident among the African Americans. Among African Americans, attitude towards eating a healthy meal was related to their BMI. As such one must focus on beliefs when developing a nutrition/health plan for the Hispanic population while concentrating on the nutritional attitudes among African Americans. Nutrition education geared towards overweight/obese individuals must be practical and aim at behavior changes. Among the Hispanic group, health/nutrition educators can focus on some basic educational topics such as the benefits of fiber and reading food labels to aid them in selecting healthy foods at the grocery stores. The least reported barrier in both ethnicities was the dislike of healthy foods. Therefore, education programs can focus on teaching diverse groups simple and quick healthy meal preparation and purchasing seasonal foods for a lower price.

## Figures and Tables

**Table 1:** Composition of food groups.

Food group	Composition	Recommended daily servings based on the food guide pyramid
Dairy	Milk Cheese Yogurt	2-3

Fruits	Fruits (excluding juices)	2–4

Vegetables	Starchy vegetables (potatoes) Green leafy vegetables Lettuce, tomato	3–5

Meats/proteins	Legumes Meats Eggs Fish and shell fish	2-3

Grains	Pasta Tortilla Bread Cereal Snacks	6–11

**Table 2:** Demographic characteristics of African American and Hispanic women.

Variable	African Americans (*N* = 92)	Hispanics (*N* = 272)
Age (years)	28.8 ± 9.1	29.2 ± 6.6
Monthly income ($)	1332.9 ± 728.4	685.0 ± 779.8
Family size	4.3 ± 1.7	5.1 ± 1.5

	%	%

Head of household	87.0	18.4
WIC participation		
Respondent	71.6	98.9
Child	52.2	67.5
SNAP participation	84.8	20.3
Work first participation	56.5	6.5
Education		
Eighth grade or less	2.2	58.5
Some high school	22.8	19.9
High school graduate or more	75.0	19.8
No response	0.0	1.9
Employment status		
Employed full-time	19.8	21.4
Employed part-time	12.1	10.3
Full time homemaker/caretaker	51.6	60.1
Unemployed	16.5	2.6
Access to transportation		
Yes, whenever	68.1	62.8
Most of the time	23.1	37.2
Very limited	8.8	0.0

**Table 3:** Levels of food security among African American and Hispanic women.

Variable	African Americans % (*n*) (*N* = 92)	Hispanics % (*n*) (*N* = 272)
Food secure/marginal food secure	57.6 (53)	14.3 (39)
Low food security	25.0 (23)	77.2 (210)
Very low food security	17.4 (16)	8.5 (23)

**Table 4:** BMI classifications of African American and Hispanic women.

Variable	African Americans % (*n*) (*N* = 92)	Hispanics % (*n*) (*N* = 272)
Underweight (<18.5)/normal (18.5–24.99)	20.9 (19)	21.6 (59)
Overweight (≥25)	17.6 (16)	38.1 (104)
Obese (≥30)	61.5 (57)	40.3 (109)

**Table 5:** Dietary intake of food groups among African American and Hispanic women.

Food group	African Americans Mean ± SD (range) (*N* = 92)	Hispanics Mean ± SD (range) (*N* = 272)
Dairy	1.58 ± 1.46 (0–7.57)	2.08 ± 1.23 (0–7)
Fruit	0.75 ± 1.61 (0–5)	1.17 ± 1.66 (0–7)
Vegetables	0.71 ± 0.96 (0.03–7.57)	0.50 ± 0.66 (0–8)
Meat/beans	1.64 ± 1.24 (1.14–6)	1.98 ± 1.14 (0.43–11)
Grains	2.52 ± 2.36 (0.1–17.57)	3.17 ± 1.70 (0.2–17)

Number of dietary recommendations met	% (*n*)	% (*n*)

0	43.5 (40)	25.2 (68)
1	31.5 (29)	30 (81)
2	13 (12)	27 (73)
3	9.8 (9)	15.6 (42)
4	2.2 (2)	1.9 (5)
5	0 (0)	0.4 (1)

Mean dietary intake	Mean ± SD	Mean ± SD

	0.192 ± 0.216	0.286 ± 0.225
